# Vulnerable Coronary Plaques on CT Angiography: Imaging Biomarkers, Artificial Intelligence, and Emerging Technologies for Risk Stratification

**DOI:** 10.3390/medsci14060639

**Published:** 2026-10-05

**Authors:** Alin Horatiu Nedelcu, Emil Anton, Ancuta Lupu, Razvan Tudor Tepordei, Simona Alice Partene Vicoleanu, Ana Maria Dumitrescu, Dragos Valentin Crauciuc, Valeriu Aurelian Chirica, Ionela Daniela Morariu, Vasile Valeriu Lupu, Cristinel Ionel Stan, Marius Constantin Moraru, Cosmin Gabriel Popa, Delia Lidia Salaru, Manuela Ursaru

**Affiliations:** 1 Grigore T. Popa University of Medicine and Pharmacy, 16 Universitatii Street, 700115 Iasi, Romania; alin.nedelcu@umfiasi.ro (A.H.N.); emil.anton@yahoo.com (E.A.); razvan.tepordei@umfiasi.ro (R.T.T.); partene.vicoleanu@umfiasi.ro (S.A.P.V.); ana-maria_dumitrescu@umfiasi.ro (A.M.D.); dragos.crauciuc@umfiasi.ro (D.V.C.); valeriu-aurelian.chirica@umfiasi.ro (V.A.C.); ionela.morariu@umfiasi.ro (I.D.M.); vasile.lupu@umfiasi.ro (V.V.L.); cristinel.stan@umfiasi.ro (C.I.S.); marius.moraru@umfiasi.ro (M.C.M.); cosmin-gabriel.popa@umfiasi.ro (C.G.P.); delia.salaru@umfiasi.ro (D.L.S.); manuela.ursaru@umfiasi.ro (M.U.); 2 Radiology Clinic, Recovery Hospital, 700661 Iasi, Romania; 3 Radiology Clinic, “Sf Spiridon” County Clinical Emergency Hospital, 700111 Iasi, Romania

**Keywords:** coronary CT angiography, vulnerable plaque, coronary artery disease, positive remodeling, spotty calcifications, napkin-ring sign, fat attenuation index, artificial intelligence

## Abstract

Background: Coronary artery disease remains the leading cause of morbidity and mortality worldwide. Increasing evidence has demonstrated that many acute coronary events originate not from severely obstructive lesions but from structurally unstable atherosclerotic plaques. Consequently, the identification of vulnerable plaques has become a major objective in contemporary cardiovascular imaging. Coronary computed tomography angiography (CCTA) has evolved from a modality primarily focused on luminal stenosis assessment into a comprehensive non-invasive technique capable of evaluating plaque morphology, composition, and overall coronary atherosclerotic burden. Results: Our review summarizes current evidence regarding the role of CCTA in the detection and characterization of vulnerable coronary plaques. Particular emphasis is placed on imaging biomarkers associated with plaque instability, including low-attenuation plaque, positive remodeling, spotty calcifications, and the napkin-ring sign, which have been consistently linked to an increased risk of adverse cardiovascular events. Advances in quantitative plaque analysis have further enhanced the ability of CCTA to assess plaque burden and monitor disease progression. Emerging technologies are rapidly expanding the capabilities of coronary CT imaging. Spectral CT techniques and photon-counting detector CT offer improved tissue characterization and enhanced visualization of plaque components. In parallel, artificial intelligence and machine learning algorithms are enabling automated plaque detection, segmentation, and risk prediction, facilitating more precise and reproducible analysis of large imaging datasets. Conclusions: Despite these advances, limitations remain, including restricted spatial resolution for microstructural plaque assessment and the limited ability of conventional CT to directly evaluate biological processes such as inflammation. Future developments integrating advanced CT technologies, artificial intelligence, and multimodality imaging are expected to further improve the non-invasive identification of vulnerable plaques and personalized cardiovascular risk stratification.

## 1. Introduction

Coronary artery disease (CAD) remains the leading cause of morbidity and mortality worldwide, accounting for a substantial proportion of cardiovascular deaths despite significant advances in prevention, diagnosis, and treatment [1,2,3,4,5,6]. Traditionally, the assessment of CAD has focused primarily on the degree of luminal stenosis; however, accumulating evidence has demonstrated that acute coronary events frequently originate from plaques that do not necessarily produce severe luminal obstruction. Instead, plaque composition and biological activity play a critical role in determining plaque instability and the risk of rupture [7,8,9].

The concept of the vulnerable plaque has therefore emerged as a central paradigm in contemporary cardiovascular research. Vulnerable plaques are typically characterized by a large lipid-rich necrotic core, a thin fibrous cap, active inflammation, and positive vascular remodeling. These structural and biological features predispose the plaque to rupture or erosion, triggering thrombosis and potentially leading to acute coronary syndromes. Consequently, the identification of high-risk plaque features before the occurrence of clinical events represents a major objective in modern cardiovascular imaging [8,10,11,12,13].

In recent years, coronary computed tomography angiography (CCTA) has evolved from a purely anatomical imaging modality used to assess luminal stenosis into a powerful non-invasive tool capable of characterizing atherosclerotic plaque morphology. Advances in CT technology and image post-processing have enabled the identification of several imaging biomarkers associated with plaque vulnerability, including low-attenuation plaque, positive remodeling, spotty calcifications, and the napkin-ring sign. Numerous clinical studies have demonstrated that these features are associated with an increased risk of future adverse cardiovascular events [7,8,14,15,16].

Beyond qualitative assessment, emerging quantitative approaches and artificial intelligence-based tools are further enhancing the ability of CCTA to evaluate plaque burden, composition, and progression. These developments position coronary CTA as a promising modality not only for the diagnosis of CAD but also for risk stratification and personalized management of patients with atherosclerotic disease [17,18].

The aim of this review is to summarize the current knowledge regarding the assessment of vulnerable plaques using coronary CT angiography, focusing on imaging biomarkers, quantitative plaque analysis, clinical implications, and future directions in this rapidly evolving field.

## 2. Literature Search Strategy

To ensure a comprehensive and up-to-date review of the literature, we conducted a systematic search of two major biomedical databases: PubMed and Web of Science. The search strategy was designed to identify both broad, high-impact publications addressing the overall topic of coronary computed tomography angiography (CCTA) and vulnerable plaque assessment, as well as more focused studies relevant to each of the specific sections discussed in this review.

The literature search incorporated a combination of Medical Subject Headings (MeSH), free-text terms, and Boolean operators. The primary keywords included coronary CT angiography, vulnerable plaque, coronary artery disease, positive remodeling, spotty calcifications, napkin-ring sign, fat attenuation index, and artificial intelligence. Representative search queries included: (“coronary CT angiography” [All Fields]) AND (“artificial intelligence” [All Fields]), together with multiple combinations of the aforementioned keywords to maximize sensitivity while maintaining relevance.

The retrieved records were screened for relevance based on their scientific quality, methodological rigor, clinical significance, and contribution to the understanding of vulnerable coronary plaque imaging and artificial intelligence applications in CCTA. Particular emphasis was placed on original research articles, systematic reviews, meta-analyses, and consensus or guideline documents published in peer-reviewed journals.

Following the evaluation, 185 references were selected for inclusion in this review. These studies were considered the most relevant and representative of the current state of knowledge and provided the scientific foundation for the topics discussed throughout the manuscript.

## 3. Pathophysiology of Vulnerable Plaques

Atherosclerosis is a chronic, multifactorial inflammatory disease of the arterial wall characterized by lipid accumulation, endothelial dysfunction, and progressive structural remodeling. The development of vulnerable plaques represents the culmination of a complex interaction between metabolic, inflammatory, gut microbiota and mechanical processes that ultimately predispose the lesion to rupture or erosion, leading to thrombotic events and acute coronary syndromes [19,20,21,22,23].

Congenital coronary artery malformations and atherosclerotic plaque formation are linked primarily through their effects on coronary hemodynamics and vascular biomechanics. Variations in coronary anatomy, such as anomalous origins, abnormal vessel angulation, myocardial bridging, or interarterial courses, can alter blood flow patterns within the coronary circulation. These alterations may produce regions of low or oscillatory wall shear stress, turbulent flow, or localized mechanical stress on the arterial wall. Such hemodynamic conditions are known to promote endothelial dysfunction, lipid accumulation, and inflammatory activity, which represent key mechanisms in the development of atherosclerotic plaques [24,25,26].

In their study including 5641 patients, among whom 467 were identified with coronary artery bifurcation anomalies, Ziyrek et al. [26] demonstrated a statistically significant association between this anatomical variation and the formation of atherosclerotic plaques. Moreover, a bifurcation angle greater than 80.5° was associated with significant stenosis within the first 5 mm distal to the bifurcation. Similarly, abnormal vessel angulation and variations in coronary origin may also influence plaque distribution. Acute take-off angles or anomalous coronary origins can produce flow disturbances and areas of reduced shear stress that predispose the vessel wall to atherosclerotic changes. These hemodynamic alterations may contribute not only to plaque formation but also to the development of plaques with features of vulnerability.

Certain coronary anomalies may predispose specific vascular segments to localized plaque development, particularly in areas where abnormal vessel geometry modifies flow dynamics. For example, myocardial bridging has been associated with accelerated plaque formation in the proximal arterial segment, while abnormal coronary origins or sharp vessel angulations may create regions susceptible to atherogenesis [24,25].

Computed tomography angiography (CTA) plays an important role in the evaluation of vascular anatomy, as it enables comprehensive, non-invasive visualization of arterial morphology, vessel geometry, and associated pathological changes. Owing to its high spatial resolution and three-dimensional reconstruction capabilities, CTA has become a valuable imaging modality for the assessment of a wide spectrum of vascular malformations and anatomical variants, allowing detailed analysis of vessel origin, course, branching patterns, and structural abnormalities.

Within this broader context, coronary CT angiography (CCTA) represents a specialized application of CTA that allows simultaneous evaluation of coronary artery anatomy, vessel geometry, and atherosclerotic plaque characteristics. By providing high-resolution three-dimensional visualization of the coronary tree, CCTA enables detailed assessment of anatomical variations, bifurcation angles, and plaque morphology. This integrated approach facilitates a better understanding of the relationship between coronary artery anatomy, local hemodynamic conditions, and the development of vulnerable atherosclerotic plaques [27,28,29,30].

Hemodynamic forces play a crucial role in the initiation and progression of atherosclerosis. Several local flow-related factors, including wall shear stress and vascular geometry, influence endothelial function and plaque development. The main hemodynamic determinants involved in coronary plaque formation are summarized in Table 1.

Future research combining CCTA-derived anatomical analysis with computational fluid dynamics and artificial intelligence may further clarify the role of coronary geometry in plaque development and progression.

### 3.1. Endothelial Dysfunction and Early Atherogenesis

The earliest stage of atherosclerosis is initiated by endothelial dysfunction, which increases vascular permeability to circulating lipoproteins and promotes leukocyte adhesion to the vascular wall. Low-density lipoproteins (LDLs) infiltrate the intima and undergo oxidative modification, triggering an inflammatory response. Monocytes migrate into the arterial wall and differentiate into macrophages, which internalize oxidized LDL through scavenger receptors, forming lipid-laden foam cells. The accumulation of foam cells and extracellular lipid deposits results in the formation of fatty streaks, representing the earliest visible lesions of atherosclerosis [43,44].

### 3.2. Formation of the Lipid-Rich Necrotic Core

As the atherosclerotic process progresses, continued lipid accumulation and macrophage apoptosis contribute to the formation of a lipid-rich necrotic core, a hallmark of vulnerable plaques. Macrophages and activated T lymphocytes release inflammatory cytokines and proteolytic enzymes, including matrix metalloproteinases (MMPs), which degrade extracellular matrix components within the plaque. This process weakens the structural integrity of the fibrous cap and promotes plaque instability [44,45,46].

The necrotic core enlarges through ongoing lipid deposition and defective clearance of apoptotic cells, a process known as impaired efferocytosis. This progressive expansion increases mechanical stress within the plaque and contributes to the destabilization of the overlying fibrous cap [47].

### 3.3. Thin-Cap Fibroatheroma and Plaque Instability

The morphological substrate most commonly associated with plaque rupture is the thin-cap fibroatheroma (TCFA). This lesion is characterized by a large necrotic lipid core covered by a thin fibrous cap, typically infiltrated by macrophages and inflammatory cells. These cells produce enzymes that degrade collagen and inhibit smooth muscle cell-mediated matrix synthesis, leading to progressive thinning of the fibrous cap [47,48].

Mechanical forces, including shear stress and pulsatile pressure, further increase the vulnerability of these plaques. When the fibrous cap becomes critically thin, even minor mechanical stress may result in plaque rupture, exposing thrombogenic material from the necrotic core to the circulating blood [47].

### 3.4. Plaque Rupture, Erosion, and Thrombosis

Two principal mechanisms are responsible for the majority of acute coronary events: plaque rupture and plaque erosion. Plaque rupture occurs when the fibrous cap tears, allowing highly thrombogenic components of the necrotic core to come into direct contact with circulating platelets and coagulation factors. This triggers rapid thrombus formation, which may partially or completely occlude the coronary artery [47,48,49,50,51,52].

Plaque erosion, although less frequently associated with large necrotic cores, involves endothelial denudation without cap rupture and also leads to thrombosis. Both mechanisms contribute to acute coronary syndromes and highlight the importance of identifying plaques with features of vulnerability before the occurrence of clinical events [49,51,53].

### 3.5. Vascular Remodeling and Plaque Progression

An additional important aspect of plaque vulnerability is positive vascular remodeling, a compensatory enlargement of the vessel wall that preserves the luminal diameter despite increasing plaque burden. While this process initially maintains coronary blood flow, it may conceal significant atherosclerotic disease on conventional angiography. Importantly, positively remodeled plaques are frequently lipid-rich and inflamed, making them more prone to rupture [44,53,54,55].

Understanding the biological mechanisms underlying plaque vulnerability is essential for interpreting imaging biomarkers identified by coronary CT angiography. Many of the CT-derived features associated with high-risk plaques, such as low-attenuation plaque, positive remodeling, and spotty calcifications, reflect these underlying pathological processes and therefore serve as non-invasive surrogates of plaque instability.

## 4. Imaging Modalities for Plaque Assessment

Accurate assessment of atherosclerotic plaque morphology and vulnerability is essential for identifying patients at increased risk of acute coronary events. Over the past two decades, several imaging techniques have been developed to characterize coronary plaques, ranging from invasive catheter-based methods to non-invasive imaging modalities. Each technique provides distinct information regarding plaque structure, composition, and biological activity, contributing to a more comprehensive understanding of coronary atherosclerosis.

### 4.1. Invasive Imaging Techniques

Invasive coronary imaging modalities remain the reference standard for detailed plaque characterization due to their high spatial resolution and ability to visualize microstructural features of the arterial wall.

Intravascular ultrasound (IVUS) is widely used to evaluate plaque burden and vessel remodeling. By generating cross-sectional images of the coronary artery using ultrasound waves, IVUS enables visualization of the vessel wall and measurement of plaque volume. It is particularly useful for detecting positive remodeling and assessing the overall extent of atherosclerotic disease [56,57]. Recent technological advancements have significantly expanded the capabilities of intravascular ultrasound imaging. Improvements in catheter design, image resolution, and signal processing have enhanced the ability of IVUS to provide detailed visualization of the coronary vessel wall. As a result, IVUS enables comprehensive characterization of intracoronary pathology, including plaque morphology, plaque burden, and vessel wall architecture. As highlighted by Truesdell et al. [58] in his review, these technical developments have strengthened the role of IVUS as a valuable intravascular imaging modality for the assessment of coronary atherosclerosis and plaque characteristics.

Optical coherence tomography (OCT) provides significantly higher spatial resolution than intravascular ultrasound (IVUS), enabling detailed visualization of plaque microstructure. With a spatial resolution up to ten times greater than that of IVUS, OCT allows precise assessment of key features such as fibrous cap thickness, macrophage infiltration, microchannels, and plaque rupture [58]. Because of its high resolution, OCT is considered one of the most accurate methods for identifying thin-cap fibroatheromas, which represent the morphological substrate of vulnerable plaques [57,59].

Another topic of growing interest, also highlighted by Kageyama et al. [57], is the combined use of IVUS and OCT imaging. The integration of these two modalities allows complementary assessment of coronary plaques, combining the deeper vessel wall penetration of IVUS with the superior spatial resolution of OCT. Additionally, angiographic registration is possible with dedicated systems, enabling more precise mapping of OCT findings onto the coronary angiogram. Artificial intelligence–based methodologies have also been developed to assist in the detection and quantification of coronary calcifications and to support treatment planning algorithms. Furthermore, hybrid IVUS–OCT systems have recently been developed with the aim of integrating both imaging techniques within a single catheter, thereby allowing simultaneous evaluation of plaque morphology and vessel wall architecture (Table 2).

Another invasive modality, near-infrared spectroscopy (NIRS), is designed to detect lipid-rich plaques by analyzing the spectral properties of coronary tissues [60]. NIRS is often combined with IVUS, providing complementary information about plaque composition and lipid core burden. Waksman et al. [61] and Nakagawa et al. [60] have demonstrated that plaques with large lipid cores identified by NIRS are associated with an increased risk of future vascular events.

### 4.2. Non-Invasive Imaging Techniques

Non-invasive imaging techniques have gained increasing importance due to their ability to evaluate coronary atherosclerosis without the risks associated with invasive procedures.

Coronary computed tomography angiography (CCTA) has emerged as a powerful non-invasive modality capable of assessing both coronary lumen and plaque morphology. Advances in CT technology now allow the identification of several imaging biomarkers associated with plaque vulnerability, including low-attenuation plaque, positive remodeling, spotty calcifications, and the napkin-ring sign. These features have been shown to correlate with histopathological characteristics of unstable plaques and with an increased risk of adverse cardiovascular events [62].

Beyond qualitative assessment, CCTA also enables quantitative plaque analysis, including measurement of plaque volume, composition, and remodeling index [63]. Recent developments in automated software and artificial intelligence-based analysis further enhance the ability of CCTA to detect high-risk plaques and monitor disease progression [17,64].

Other non-invasive imaging techniques are also being investigated for plaque characterization. Cardiac magnetic resonance imaging (CMRI) can provide information about vessel wall structure and plaque composition without ionizing radiation, although its spatial resolution remains limited for coronary imaging [65]. Positron emission tomography (PET) allows the assessment of metabolic and inflammatory activity within atherosclerotic plaques by using specific radiotracers, such as ^18F-FDG or ^18F-NaF, which highlight inflammatory processes and microcalcification [66,67].

### 4.3. Comparative Perspective

While invasive imaging techniques provide unparalleled spatial resolution and detailed plaque characterization, their use is limited to patients undergoing coronary angiography. In contrast, non-invasive modalities, particularly coronary CT angiography, offer the advantage of evaluating coronary anatomy and plaque features in a broader patient population. As a result, CCTA has become an essential tool for the non-invasive identification of high-risk plaques and the stratification of cardiovascular risk, bridging the gap between anatomical imaging and functional assessment of coronary artery disease.

#### 4.3.1. Comparative Evaluation of CCTA, Cardiac MRI, and PET in Plaque Assessment

Non-invasive imaging techniques have gained increasing importance in the evaluation of coronary atherosclerosis and plaque vulnerability. Among these, coronary computed tomography angiography (CCTA), cardiac magnetic resonance imaging (CMRI), and positron emission tomography (PET) provide complementary information regarding plaque morphology, composition, and biological activity. While each modality has specific strengths and limitations, their combined use has the potential to significantly improve cardiovascular risk stratification.

##### Coronary Computed Tomography Angiography (CCTA)

CCTA is currently the most widely used non-invasive technique for the anatomical evaluation of coronary arteries. It offers high spatial resolution and excellent visualization of the coronary lumen and vessel wall, allowing detailed assessment of plaque morphology and composition. One of the major advantages of CCTA is its ability to identify several imaging biomarkers associated with vulnerable plaques, including low-attenuation plaque, positive remodeling, spotty calcifications, and the napkin-ring sign. These features have been correlated with histopathological findings such as lipid-rich necrotic cores and thin-cap fibroatheromas [62].

In addition to qualitative assessment, CCTA allows quantitative plaque analysis, including measurement of plaque volume, plaque burden, and remodeling index. In their study, Bell et al. [63] demonstrated that quantitatively assessed plaque volumes are associated with an increased risk of major adverse cardiovascular events (MACE) in patients undergoing coronary CT angiography for stable coronary artery disease. However, further research involving diverse patient populations and standardized analytical methodologies is necessary to better define the clinical value and applicability of plaque quantification in routine clinical practice.

Furthermore, in their meta-analysis, Iraqi et al. [68] demonstrated that quantitative plaque assessment derived from coronary CT angiography provides significant incremental value in the evaluation of coronary artery disease. Specifically, the quantification of total plaque burden and the identification of high-risk plaque characteristics were found to be associated with myocardial ischemia and to significantly improve the discrimination of ischemia-producing coronary lesions, independently of the degree of luminal stenosis.

These findings further support the concept that the functional significance of coronary lesions cannot be determined solely on the basis of stenosis severity. Instead, the overall plaque burden and specific morphological features indicative of plaque vulnerability contribute substantially to the hemodynamic impact of a lesion. By enabling comprehensive evaluation of both plaque volume and plaque composition, CCTA-derived quantitative analysis provides a more refined assessment of lesion-specific risk. Consequently, integrating plaque quantification with traditional stenosis assessment may improve the identification of clinically relevant coronary lesions and enhance risk stratification in patients with suspected or established coronary artery disease.

Recent advances in image processing and artificial intelligence-based software have further enhanced automated plaque characterization and risk prediction. Another important advantage of CCTA is its wide availability and relatively short acquisition time, making it suitable for large-scale clinical evaluation.

However, CCTA has certain limitations. Its ability to assess plaque microstructure, such as fibrous cap thickness or macrophage infiltration, remains limited due to spatial resolution constraints. Furthermore, heavily calcified plaques may generate blooming artifacts, potentially affecting accurate plaque characterization [69,70]. Despite these limitations, CCTA remains the reference non-invasive technique for anatomical assessment of coronary atherosclerosis.

Dual-energy CT/Spectral CT and advanced plaque characterization

Recent technological developments in computed tomography have led to the emergence of spectral CT techniques, including dual-energy CT and photon-counting CT, which provide additional information about tissue composition beyond conventional attenuation-based imaging. These technologies enable improved differentiation of plaque components and may enhance the non-invasive identification of vulnerable coronary plaques [17,18].

Spectral CT operates by acquiring imaging data at different X-ray energy levels, allowing the analysis of energy-dependent attenuation properties of tissues. Several technological approaches are currently used to achieve spectral imaging, including dual-source CT systems, rapid kVp switching, dual-layer detector systems, and more recently photon-counting detector CT (PCCT). By exploiting the spectral differences in attenuation, these systems enable material decomposition and more accurate characterization of coronary plaque composition [71,72,73,74].

One of the main advantages of spectral CT is its ability to improve the identification of non-calcified plaque components, which are closely associated with plaque vulnerability. Through material decomposition algorithms, spectral CT can facilitate differentiation between lipid-rich necrotic cores, fibrous tissue, and calcified components. This capability is particularly relevant for the detection of lipid-rich plaques, which represent one of the key substrates of thin-cap fibroatheroma [74,75].

Another important feature of spectral imaging is the generation of virtual monoenergetic images (VMI) at different energy levels. Low-energy reconstructions enhance iodine contrast and improve visualization of the coronary lumen, while higher-energy images help reduce artifacts produced by dense calcifications or metallic stents. This capability may improve plaque visualization in heavily calcified coronary segments, where blooming artifacts often limit conventional CCTA assessment [71,76,77,78,79].

In their study conducted in patients with normal body mass index, Yi et al. [71] demonstrated that low-energy monoenergetic spectral reconstructions (40–50 keV) derived from 120 kVp coronary CT angiography performed on dual-layer spectral detector CT systems provide superior coronary image quality compared with conventional reconstructions obtained from low tube voltage (100 kVp) acquisitions. Moreover, this approach enables a reduction in both contrast media volume and radiation exposure while maintaining or even improving diagnostic image quality.

Spectral CT also enables iodine mapping, allowing quantitative evaluation of iodine distribution within tissues. In the context of coronary plaque imaging, iodine maps may provide indirect information regarding plaque vascularization and inflammatory activity. Increased iodine uptake within plaques may reflect the presence of vasa vasorum proliferation, which has been associated with plaque instability and inflammatory processes [80,81].

Another important application of spectral CT is the ability to generate virtual non-contrast (VNC) images for coronary artery calcium (CAC) scoring. This technique allows calcium quantification to be performed without the need for a separate non-contrast acquisition, thereby reducing both examination time and patient radiation exposure. However, as demonstrated by Pérez-Cervera et al. [82] in a study including 174 patients, body mass index significantly influences the accuracy of VNC-derived CAC measurements. Their findings showed substantial agreement between VNC-based and conventional CAC scoring in non-obese individuals, whereas the performance of VNC CAC was considerably reduced in patients with a BMI greater than 40 kg/m^2^.

Among emerging technologies, photon-counting CT represents a particularly promising advancement. Spectral photon-counting computed tomography (SPCCT) has the potential to significantly transform current imaging paradigms. This emerging technology is based on the use of advanced energy-resolving detectors known as photon-counting detectors (PCDs), which provide several advantages over conventional energy-integrating detectors (EIDs), particularly in improving the quality of virtual monoenergetic imaging (VMI). Compared with traditional detectors, PCDs are less susceptible to electronic noise and can be designed in a more compact configuration. This allows the implementation of ultra-high-resolution (UHR) imaging modes, enabling spatial resolutions that are two to three times higher than those achievable with conventional CT systems, reaching values of up to approximately 250 µm. As a result, spectral reconstructions, including virtual monoenergetic images and iodine maps, can be generated in ultra-high-resolution mode, thereby enhancing tissue characterization and diagnostic image quality. These characteristics may allow improved visualization of small coronary structures and better detection of features such as microcalcifications and lipid-rich plaque components, both of which are relevant markers of plaque vulnerability [83,84].

One of the major advantages of photon-counting detector CT is its intrinsic spectral imaging capability, which enables the routine generation of virtual monoenergetic images (VMIs) through material decomposition, typically separating iodine from soft tissue components. VMIs simulate images acquired with a monochromatic X-ray beam at a selected energy level (keV), allowing iodine attenuation values to remain independent of the tube voltage (kVp) used during image acquisition. This spectral approach improves image consistency and facilitates the standardization of quantitative measurements across different acquisition protocols and scanner settings [85,86].

For coronary artery calcium (CAC) assessment, however, careful selection of the virtual monoenergetic energy level is essential to ensure compatibility with the Agatston scoring methodology established for conventional energy-integrating detector CT. Phantom experiments performed on cadaveric hearts using a prototype PCD-CT system demonstrated that 72 keV VMIs achieved the closest agreement with calcium scores obtained from conventional 120 kVp acquisitions, supporting the use of optimized monoenergetic reconstructions for maintaining consistency with current clinical calcium scoring standards while preserving the advantages of spectral imaging [87].

In their metanalysis, Rønning et al. [87] indicate that photon-counting CT (PCCT) provides coronary artery calcium scoring (CACS) accuracy comparable to conventional energy-integrating detector CT (EID-CT), while offering important technical advantages. Prospective studies have consistently demonstrated excellent agreement between PCCT and EID-CT for Agatston score quantification, confirming that the transition to photon-counting technology does not compromise the reliability of CACS measurements. In addition, PCCT achieves significantly higher contrast-to-noise ratios and enables substantial radiation dose reduction without loss of diagnostic accuracy [88,89,90]. Collectively, these findings suggest that PCCT has the potential to become the preferred imaging platform for CACS assessment, particularly in screening and longitudinal follow-up settings where minimizing radiation exposure while maintaining reproducible calcium quantification is of paramount importance.

Although spectral CT techniques are still undergoing clinical validation, early studies suggest that these technologies may significantly enhance the ability of coronary CT angiography to characterize plaque composition and identify high-risk lesions. As spectral imaging continues to evolve, it is expected to play an increasingly important role in the non-invasive evaluation of coronary atherosclerosis and personalized cardiovascular risk stratification.

##### Cardiac Magnetic Resonance Imaging (CMRI)

Cardiac magnetic resonance imaging provides excellent soft-tissue contrast and allows detailed evaluation of the vessel wall without exposure to ionizing radiation. CMRI has the advantage of characterizing different plaque components based on tissue-specific signal properties. Using specialized sequences, MRI can identify features such as lipid-rich necrotic cores, intraplaque hemorrhage, and fibrous tissue [91,92].

Another important strength of MRI is its ability to provide simultaneous functional and tissue characterization, including myocardial perfusion, myocardial viability, and ventricular function. This makes CMRI particularly useful for comprehensive cardiovascular evaluation [92].

Despite these advantages, the use of MRI in coronary plaque imaging remains limited. The small caliber of coronary arteries, cardiac motion, and respiratory movement present technical challenges that reduce spatial resolution compared with CT [93]. As a result, MRI is currently more commonly used for imaging carotid and large-vessel atherosclerosis rather than coronary plaques. Furthermore, cardiac magnetic resonance imaging (CMRI) represents a key diagnostic modality for the detection of myocardial ischemia and the assessment of myocardial viability, offering valuable information that contributes significantly to clinical decision-making and the optimization of therapeutic strategies [92]. Ongoing technological advances, including higher magnetic field strengths and improved motion correction techniques, may improve its future role in coronary plaque assessment.

##### Positron Emission Tomography (PET)

PET imaging provides a unique advantage by allowing functional and molecular assessment of atherosclerotic plaques. Unlike CT and MRI, which primarily evaluate structural characteristics, PET can detect biological processes associated with plaque vulnerability, such as inflammation and active microcalcification [94].

The most commonly used radiotracer is ^18F-fluorodeoxyglucose (18F-FDG), which reflects metabolic activity and inflammation within the plaque by detecting increased glucose uptake in activated macrophages. Increased 18F-FDG uptake has been associated with plaques at higher risk of rupture. Another important tracer, ^18F-sodium fluoride (18F-NaF), identifies areas of active microcalcification, which represent an early stage of plaque destabilization [66,94,95].

One limitation of PET imaging is its relatively low spatial resolution, which makes precise localization of coronary plaques challenging. Additionally, myocardial uptake of FDG can interfere with coronary plaque visualization. To overcome these limitations, PET is frequently combined with CT or MRI in hybrid systems such as PET/CT or PET/MRI, allowing simultaneous anatomical and molecular imaging [96].

##### Integrated Perspective

From a comparative standpoint, each modality provides different yet complementary information about coronary atherosclerosis. CCTA excels in anatomical plaque characterization and detection of morphological high-risk features, making it the primary non-invasive modality for identifying vulnerable plaques. MRI offers superior soft-tissue characterization and functional cardiac assessment without radiation exposure, although its role in coronary plaque imaging remains limited by spatial resolution. PET provides unique insights into the biological activity of plaques, particularly inflammation and microcalcification, which are key processes in plaque destabilization (Table 3).

The integration of these imaging techniques, particularly through hybrid imaging approaches such as PET/CT and PET/MRI, represents a promising direction for future research. By combining anatomical, compositional, and molecular information, multimodal imaging has the potential to significantly enhance the identification of vulnerable plaques and improve the prediction of adverse cardiovascular events.

## 5. CT Features of High-Risk Plaques

Coronary computed tomography angiography (CCTA) has evolved from a technique primarily used for evaluating luminal stenosis into a powerful tool for non-invasive characterization of coronary atherosclerotic plaques. Advances in CT technology and post-processing algorithms have enabled the identification of specific imaging biomarkers that reflect underlying histopathological features associated with plaque instability. These features, commonly referred to as high-risk plaque (HRP) characteristics, have been consistently associated with an increased risk of acute coronary syndromes and adverse cardiovascular events, as Iraqi et al. [68] stated in his metanalyses.

Numerous studies, including the investigation conducted by Lee et al. [59], have demonstrated that luminal narrowing alone is not a strong predictor of cardiovascular (CV) mortality, challenging the traditional paradigm that the severity of stenosis is the main determinant of adverse cardiovascular events. Historically, the clinical assessment of coronary artery disease focused primarily on the degree of luminal obstruction, with lesions producing significant stenosis considered the principal targets for diagnostic evaluation and therapeutic intervention. However, accumulating evidence has shown that many acute coronary events originate from non-obstructive or moderately stenotic plaques rather than from severely narrowed segments.

Lee et al. [59], through analyses based on coronary CT angiography and longitudinal follow-up, showed that plaque burden and plaque composition were more closely associated with adverse cardiovascular outcomes than the degree of luminal stenosis itself. In particular, plaques characterized by features such as low attenuation, positive remodeling, and increased plaque volume were found to have a stronger relationship with cardiovascular mortality and major adverse cardiovascular events.

These findings support the concept that atherosclerotic disease should be viewed as a diffuse process affecting the vessel wall rather than solely as a luminal narrowing phenomenon. Vascular remodeling may initially compensate for plaque growth by expanding the arterial wall, thereby preserving the lumen despite significant plaque accumulation. As a result, lesions with substantial atherosclerotic burden may remain angiographically mild while still carrying a high risk of rupture [97,98,99].

Several CT-derived markers have been validated in clinical studies and correlate with pathological features of vulnerable plaques, including lipid-rich necrotic cores, thin fibrous caps, inflammatory infiltration, and positive vascular remodeling.

### 5.1. Low-Attenuation Plaque

Low-attenuation plaque represents one of the most important CT indicators of plaque vulnerability. On CCTA, this feature is typically defined by attenuation values below 30 Hounsfield Units (HU) within the non-calcified plaque component. Low attenuation reflects the presence of a lipid-rich necrotic core, which is a key pathological feature of unstable plaques [66,100].

Histopathological studies have demonstrated a strong correlation between low CT attenuation values and lipid-rich plaque composition. Clinically, plaques with low attenuation have been associated with a significantly increased risk of future acute coronary events. In particular, the presence of low-attenuation plaque combined with other high-risk features markedly increases the likelihood of plaque rupture [101,102].

Quantitative plaque analysis can further refine this assessment by measuring the volume of low-attenuation components, which may serve as an important marker of plaque progression and vulnerability.

### 5.2. Positive Remodeling

Positive remodeling refers to compensatory enlargement of the vessel wall at the site of atherosclerotic plaque formation, which helps maintain luminal diameter despite increasing plaque burden. This process results from inflammatory-mediated degradation of the vascular extracellular matrix and structural changes in the arterial wall [103].

On CCTA, positive remodeling is assessed using the remodeling index (RI), defined as the ratio between the vessel diameter at the plaque site and the reference vessel diameter. The study conducted by Motoyama et al. [104] established a threshold for the remodeling index, demonstrating that an RI greater than 1.1 is generally considered indicative of positive vascular remodeling.

Positively remodeled plaques are frequently associated with lipid-rich composition, inflammatory activity, and thin fibrous caps. Importantly, this phenomenon may mask the true severity of atherosclerotic disease because luminal narrowing can remain minimal despite substantial plaque burden. Numerous studies have demonstrated that plaques exhibiting positive remodeling are strongly associated with an increased risk of acute coronary events [103,104,105,106].

### 5.3. Napkin-Ring Sign (NRS)

Many scholars, such as Abdelrahman [62], Cury et al. [99], Williams et al. [103] or Kim and Choi [105], consider the napkin-ring sign, also called “ring-like sign”, one of the most specific CT imaging markers of vulnerable plaques. Moreover, Otsuka et al. [107] in their study that included 895 patients demonstrated a high association between napkin-ring sign and acute coronary events, even in the absence of other signs such as positive remodeling or low-attenuation plaque. This feature appears as a central area of low attenuation surrounded by a ring-like area of higher attenuation within a non-calcified plaque.

Pathologically, this imaging pattern corresponds to a large lipid-rich necrotic core covered by a thin fibrous cap. The outer hyperattenuating rim likely represents fibrous tissue surrounding the necrotic center [62]. Conversely, some researchers believe that hyperattenuating rim is determined by the presence of microcalcifications [108,109].

Several studies have demonstrated that the napkin-ring sign has high specificity for identifying thin-cap fibroatheromas, which are strongly associated with plaque rupture. In their study of 273 patients with acute coronary syndrome or stable angina pectoris (SAP), Kashiwagi et al. [110] identified an association of napkin-ring sign with fibroatheroma. Although the study has limitations represented by the small number of patients, as well as the unequal ratio of patients with ACS and SAP, it demonstrated a higher frequency of napkin-ring sign in patients with ACS in both the case of culprit lesions and non-culprit lesions.

An important feature of lesions exhibiting the napkin-ring sign is their dynamic evolution over time. Brutkiewicz et al. [111] reported a higher prevalence of such lesions in patients considered to be at increased cardiovascular risk. Furthermore, their findings indicated that diabetes mellitus represents an important factor associated with both the progression of atherosclerotic plaques and an increased number of lesions displaying the napkin-ring sign.

Although the napkin-ring sign is less frequently observed than other high-risk features, its presence is considered a strong predictor of future coronary events and is therefore highly relevant for risk stratification.

### 5.4. Perivascular Fat Attenuation Index (FAI)

Coronary artery inflammation exerts a significant influence on the biological behavior of the surrounding perivascular adipose tissue, notably by inhibiting normal adipocyte differentiation and lipid accumulation. These inflammatory signals alter the metabolic and structural properties of the adjacent perivascular fat, leading to measurable changes in its radiographic characteristics. In this context, a novel imaging biomarker known as the perivascular fat attenuation index has been developed to non-invasively assess coronary inflammation [97,112].

In their comparative study including two cohorts of 1872 and 2040 patients, respectively, Oikonomou et al. [97] demonstrated that elevated perivascular fat attenuation index values measured around the proximal segments of the right coronary artery and the left anterior descending artery were predictive of both all-cause and cardiac mortality. Furthermore, the authors reported a strong correlation between FAI measurements obtained from these two coronary territories. Importantly, the study established a threshold value of −70.1 Hounsfield units as the optimal cutoff for risk stratification. Perivascular fat attenuation values exceeding this threshold were associated with a significantly increased incidence of adverse coronary events and higher rates of cardiac mortality.

In a meta-analysis including 17 studies and a total of 10,482 patients, Tan et al. [98] emphasized the prognostic significance of the perivascular fat attenuation index in the context of coronary artery disease. Their findings demonstrated that elevated FAI values were significantly associated with the occurrence of major adverse cardiac events and with the presence of high-risk plaque features. These results further support the role of FAI as a non-invasive imaging biomarker of coronary inflammation, providing incremental prognostic information beyond conventional assessments of coronary stenosis and plaque morphology.

Yu et al. [113] investigated the association of FAI with hemodynamic refraction of stenotic lesions. In the 219 lesions studied, FAI lesions were significantly larger in the group of hemodynamically significant lesions. However, the use of FAI as a predictor of ischemic coronary stenosis requires association with other predictive factors such as stenosis diameter or total plaque volume. Similar findings were reported by Sun et al. [114] in their study involving serial coronary CT angiography performed over a 48-week period in a cohort of 252 patients with and without major adverse cardiovascular events. At baseline, perivascular fat attenuation index values were significantly higher in patients who subsequently developed MACE. During follow-up, FAI levels remained elevated in the MACE group, whereas a reduction in FAI was observed among patients who did not experience adverse events. Ultimately, the authors identified FAI, stenosis diameter, and total plaque volume as independent predictors of MACE.

Importantly, the assessment of FAI extends the capabilities of conventional CCTA beyond the structural evaluation of coronary stenosis and plaque morphology. By enabling the detection of vascular inflammation, a key driver of plaque progression and destabilization, FAI offers additional prognostic information that may help identify patients at increased risk of future cardiovascular events. Consequently, integrating perivascular fat analysis into routine CCTA interpretation may enhance cardiovascular risk stratification and contribute to more personalized management strategies in patients with coronary artery disease.

Although the Fat Attenuation Index has emerged as a promising imaging biomarker of coronary inflammation, its quantitative assessment remains susceptible to several sources of technical and biological variability. Pericoronary adipose tissue (PCAT) attenuation, expressed in Hounsfield units, is significantly influenced by CT acquisition parameters. For example, increasing the tube voltage from 70 kV to 120 kV has been shown to alter PCAT attenuation for non-contrast acquisition from −111 ± 11 HU at 55 keV to −82 ± 9 HU at 80 keV and for contrast-enhanced acquisition from −104 ± 11 HU at 55 keV to −81 ± 9 HU at 80 keV. This effect is large enough to influence risk categorization if not appropriately corrected [115]. Additional variability arises from differences in image reconstruction algorithms, scanner vendors, and acquisition protocols, highlighting the importance of standardized imaging techniques.

Beyond technical factors, patient-specific biological characteristics also affect FAI measurements. Individuals with obesity typically exhibit hypertrophic, lipid-rich adipocytes that demonstrate lower X-ray attenuation, resulting in more negative HU values, whereas lean individuals generally present with higher baseline adipose tissue attenuation [116]. Age, sex, metabolic profile, and anatomical differences among coronary arteries further contribute to the observed heterogeneity, limiting the direct comparison of absolute FAI values across different patient populations.

To overcome these limitations, an artificial intelligence-based FAI-Score has recently been developed as a standardized and clinically interpretable alternative to conventional FAI measurements [117]. The AI algorithm performs fully automated segmentation of the coronary arteries and surrounding perivascular adipose tissue, quantifies three-dimensional attenuation gradients, and subsequently corrects the measurements for multiple confounding variables, including tube voltage, patient age, sex, and artery-specific anatomical characteristics. Rather than reporting a raw attenuation value, the algorithm generates a normalized, unit-free score that can be interpreted using age- and sex-specific reference nomograms [117,118,119]. This approach substantially improves the reproducibility, inter-scanner comparability, and clinical applicability of FAI assessment.

The implementation of AI-driven standardization represents an important step toward the integration of perivascular fat imaging into routine cardiovascular practice. By minimizing technical variability and accounting for patient-specific biological differences, AI-derived FAI-Score enhances the reliability of inflammation assessment and facilitates individualized cardiovascular risk stratification, preventive interventions, and therapeutic decision-making. Continued multicenter validation and harmonization efforts will be essential to establish FAI as a robust imaging biomarker and to support its transition from a predominantly research-oriented metric to a routinely reported parameter in coronary CT angiography.

### 5.5. Spotty Calcifications

Calcification is generally considered a marker of more advanced and stable atherosclerosis; however, small focal calcifications within non-calcified plaques, known as spotty calcifications, are associated with plaque instability [66,103].

On CCTA, spotty calcifications are typically defined as small calcified deposits less than 3 mm in size embedded within non-calcified plaque components. These calcifications are thought to reflect active inflammatory processes and early stages of microcalcification occurring within unstable plaques [66,68,104].

Histological studies have suggested that spotty calcifications may increase local mechanical stress within the plaque, contributing to fibrous cap disruption. Moreover, assessment of calcium levels in atherosclerotic plaque may be a marker of vulnerable plaque, as demonstrated by Pezel et al. [120] in patients with non-ST elevation myocardial infarction. Clinically, their presence has been associated with increased risk of plaque progression and adverse cardiovascular outcomes.

### 5.6. High-Risk Plaque Phenotype

The prognostic value of CCTA increases when multiple high-risk plaque features are identified within the same lesion. Studies have demonstrated that plaques exhibiting two or more high-risk characteristics, such as low attenuation, positive remodeling, napkin-ring sign, and spotty calcifications, are associated with a significantly increased risk of future acute coronary syndromes [62,103,104,121,122].

Large clinical trials like ACCURACY [123], SCOT-HEART [124], PROSPECT [125], PROMISE [126], ROMICAT-II [127] or ICONIC [128] have shown that patients with high-risk plaque features detected on CCTA experience higher rates of major adverse cardiovascular events compared with patients without these characteristics. Consequently, the identification of HRP features has become increasingly important for risk stratification and preventive therapeutic strategies, including aggressive lipid-lowering therapy and closer clinical monitoring.

### 5.7. Quantitative Plaque Analysis

Recent advances in CT image analysis have enabled the development of semi-automated and fully automated plaque quantification tools, which allow precise measurement of plaque volume and composition. These tools can differentiate between calcified, fibrous, and lipid-rich components, providing a more objective assessment of plaque vulnerability.

Quantitative parameters such as low-attenuation plaque burden, total plaque volume, and remodeling index have shown strong predictive value for future cardiovascular events. In addition, artificial intelligence and machine learning algorithms are increasingly being applied to large imaging datasets, enabling improved detection of subtle high-risk plaque features (Table 4).

All these developments position CCTA as a comprehensive modality capable of identifying both morphological and compositional characteristics of vulnerable plaques, supporting its growing role in the early detection and management of coronary artery disease.

## 6. Clinical Implications of Vulnerable Plaque Detection on Coronary CT Angiography

The identification of high-risk plaque features on coronary computed tomography angiography has important clinical implications for the management of patients with coronary artery disease. Beyond its role in detecting luminal stenosis, CCTA provides valuable information regarding plaque morphology and composition, enabling the early identification of patients at increased risk of acute coronary events. The recognition of vulnerable plaque characteristics may therefore influence risk stratification, guide preventive therapeutic strategies, and support clinical decision-making regarding further diagnostic or invasive procedures [129].

### 6.1. Risk Stratification

One of the most important clinical applications of CCTA is its ability to improve cardiovascular risk stratification. Traditional risk assessment models are largely based on clinical risk factors and the degree of luminal stenosis. However, these approaches do not fully capture the biological behavior of atherosclerotic plaques. Numerous studies have demonstrated that adverse cardiovascular events often originate from plaques that produce only mild or moderate luminal narrowing [8,9,12,63].

CCTA allows the identification of high-risk plaque features, including low-attenuation plaque, positive remodeling, spotty calcifications, and the napkin-ring sign. The presence of these imaging biomarkers has been associated with an increased risk of major adverse cardiovascular events, even in patients without significant obstructive coronary disease. Consequently, the evaluation of plaque characteristics using CCTA may provide incremental prognostic information beyond stenosis severity alone [9,14,129].

### 6.2. Preventive Therapy

The detection of vulnerable plaque features may prompt the initiation or optimization of preventive medical therapy. Early identification of high-risk plaques allows clinicians to implement aggressive risk factor modification strategies aimed at stabilizing atherosclerotic lesions and reducing the likelihood of plaque rupture.

Preventive interventions may include lifestyle modification, improved control of cardiovascular risk factors such as hypertension and diabetes, and pharmacological therapy targeting lipid metabolism and vascular inflammation. By identifying patients with subclinical but high-risk coronary atherosclerosis, CCTA can facilitate earlier intervention and potentially reduce the incidence of acute coronary events.

### 6.3. Intensification of Lipid-Lowering Therapy

Among preventive strategies, lipid-lowering therapy, particularly statin treatment, plays a central role in plaque stabilization. Statins have been shown to reduce lipid content within plaques, decrease inflammatory activity, and promote plaque calcification, which is generally associated with increased plaque stability [130,131].

CCTA-based plaque analysis enables the detection of lipid-rich plaques and the quantification of non-calcified plaque burden. The presence of such features may justify intensification of statin therapy, including the use of high-intensity statins or additional lipid-lowering agents such as ezetimibe or PCSK9 inhibitors [132,133,134]. Serial CCTA studies have also demonstrated that intensive lipid-lowering therapy may lead to slower plaque progression and increased plaque stabilization, supporting the role of imaging-guided therapeutic strategies [131,134,135,136].

### 6.4. Guidance for Invasive Coronary Angiography

Another important clinical implication of CCTA is its potential role in guiding decisions regarding invasive coronary angiography and further functional testing. While the presence of high-risk plaque features does not necessarily indicate hemodynamically significant stenosis, these lesions may represent sites at increased risk of future rupture [137].

In patients presenting with symptoms suggestive of coronary artery disease, the detection of high-risk plaque characteristics on CCTA may support the need for closer clinical monitoring, additional functional assessment, referral for invasive coronary angiography or even coronary artery bypass grafting. Conversely, the absence of significant plaque burden or high-risk features may help avoid unnecessary invasive procedures [138,139].

### 6.5. Toward Personalized Cardiovascular Management

Overall, the integration of plaque characterization into routine CCTA evaluation represents an important step toward personalized cardiovascular risk assessment and management. By combining anatomical, compositional, and clinical information, CCTA allows clinicians to better identify patients who may benefit from intensified preventive therapy or closer surveillance, ultimately contributing to improved cardiovascular outcomes.

Several large clinical trials have demonstrated the prognostic value of coronary CT angiography in identifying high-risk plaque features associated with future cardiovascular events [66]. A summary of the most relevant studies is presented in Table 5.

## 7. Artificial Intelligence in CCTA Plaque Analysis

Artificial intelligence (AI) has emerged as a transformative tool in cardiovascular imaging, offering new possibilities for the automated assessment and characterization of coronary atherosclerotic plaques. In the context of coronary computed tomography angiography (CCTA), AI-based approaches, particularly machine learning and deep learning algorithms, have demonstrated significant potential in improving plaque detection, segmentation, quantification, and risk prediction. By enabling rapid and reproducible analysis of large imaging datasets, AI may substantially enhance the clinical value of CCTA in the evaluation of vulnerable plaques [17,64,139,140,141].

### 7.1. Automated Plaque Detection and Segmentation

One of the most important applications of AI in CCTA is the automated detection and segmentation of coronary plaques. Deep learning algorithms, particularly convolutional neural networks (CNNs), can analyze volumetric CT datasets and automatically identify coronary arteries, delineate the vessel lumen and wall, and detect atherosclerotic plaques [141,142,143,144].

Automated plaque segmentation allows precise differentiation between calcified, non-calcified, and mixed plaques, significantly reducing the time required for manual analysis. Several studies such as those conducted by Jávorszky et al. [142], Ghanem et al. [143], Kolossváry et al. [145] or Jonas et al. [146], have demonstrated that AI-assisted plaque detection achieves diagnostic performance comparable to expert readers while improving reproducibility and reducing interobserver variability.

### 7.2. Quantitative Plaque Analysis

AI-based tools enable advanced quantitative plaque characterization, providing objective measurements of plaque burden and composition. These systems can automatically calculate several clinically relevant parameters, including total plaque volume, non-calcified plaque volume, low-attenuation plaque volume, remodeling index or percent atheroma volume [147,148].

Such quantitative metrics have been shown to correlate with the risk of future cardiovascular events. Automated plaque quantification therefore facilitates longitudinal monitoring of plaque progression or regression, which may be particularly useful for evaluating the effects of medical therapies such as lipid-lowering treatment.

### 7.3. Radiomics and Texture Analysis

Radiomics represents another promising application of AI in coronary plaque imaging. Radiomics involves the extraction of large numbers of quantitative imaging features, such as texture, shape, and intensity patterns, from medical images. These features can capture subtle variations in plaque structure that may not be detectable by visual inspection [149,150,151].

Radiomic analysis of CCTA datasets has shown potential for identifying imaging patterns associated with plaque vulnerability, inflammation, and lipid-rich composition [150,152]. By integrating multiple radiomic features, machine learning models may provide more accurate prediction of plaque instability compared with conventional imaging markers alone.

### 7.4. AI-Based Risk Prediction

Beyond plaque characterization, AI algorithms are increasingly being used to develop predictive models for cardiovascular risk stratification. By combining imaging biomarkers with clinical and demographic data, machine learning systems can identify complex patterns associated with adverse cardiovascular outcomes [153].

Recent studies have demonstrated that AI-driven risk models based on CCTA-derived plaque features may outperform traditional risk assessment tools in predicting major adverse cardiovascular events (MACE). Such models may therefore support personalized treatment strategies by identifying patients who could benefit from more aggressive preventive therapies [154,155,156].

### 7.5. Integration with Advanced CT Technologies

AI applications are also being integrated with emerging CT technologies, including spectral CT and photon-counting CT, which provide enhanced plaque characterization capabilities. AI algorithms can process the large datasets generated by these advanced imaging techniques and extract clinically relevant information related to plaque composition and vulnerability. Furthermore, AI-based analysis is increasingly incorporated into fully automated CCTA workflows, enabling rapid and standardized plaque evaluation in routine clinical practice [17,18].

The integration of computational fluid dynamics, artificial intelligence, and coronary computed tomography angiography enables a comprehensive, non-invasive assessment of coronary anatomy, lesion-specific hemodynamics, and plaque characteristics. Patient-specific three-dimensional coronary models reconstructed from CCTA datasets can be used to simulate blood flow, pressure distribution, and biomechanical forces acting on the vessel wall. Artificial intelligence–based algorithms may facilitate automated coronary artery segmentation, plaque detection and quantification, image quality improvement, and the rapid estimation of hemodynamic parameters, thereby reducing processing time and interobserver variability [157,158,159].

The most established clinical application is CCTA-derived fractional flow reserve, which provides functional information regarding the ischemic significance of coronary stenoses and complements the anatomical evaluation offered by CCTA. Machine-learning and deep-learning approaches have also been developed to estimate CT-derived fractional flow reserve without the need for computationally intensive conventional CFD simulations. In addition, the integration of AI-based plaque analysis with CFD-derived parameters, including wall shear stress, axial plaque stress, pressure gradients, vorticity, and helicity, may provide further insight into plaque progression, vulnerability, and the risk of future adverse cardiovascular events [158,160,161]. Thus, the combined use of CCTA, CFD, and artificial intelligence represents a transition from purely anatomical coronary assessment toward integrated anatomical, functional, biomechanical, and predictive plaque characterization.

### 7.6. Clinical Implications and Future Perspectives of Artificial Intelligence in CCTA

Despite the promising results reported in recent studies [149,150,153,156], several challenges remain before AI-based plaque analysis becomes fully integrated into clinical practice. These include the need for large multicenter datasets for algorithm training, validation across different CT platforms, and standardized reporting frameworks.

Nevertheless, the use of artificial intelligence (AI) as a ready-for-use clinical tool should be approached with caution. The performance and generalizability of AI algorithms are highly dependent on technical acquisition parameters and, consequently, on the imaging equipment and reconstruction protocols employed. Several studies have demonstrated that radiomic features are substantially influenced by factors such as radiation dose, image reconstruction algorithms, slice thickness, kernel selection, and scanner manufacturer, potentially compromising the reproducibility and external validity of AI-based models [162,163].

To address these challenges, the Image Biomarker Standardisation Initiative (IBSI) was established with the primary objective of improving the reproducibility, robustness, and standardization of radiomic analyses across institutions and imaging platforms, thereby facilitating the clinical translation of radiomics. The initiative aims to define reference values and standardized methodologies for feature extraction, enabling meaningful comparisons among studies and enhancing confidence in AI-derived imaging biomarkers [164].

The first two completed phases of the IBSI project focused on the standardization of commonly used radiomic features (IBSI-1) and the harmonization of image preprocessing and filtering techniques employed in radiomic analyses (IBSI-2). Validation was performed through a three-stage framework comprising experiments on a digital phantom (Phase 1), CT imaging data from a patient with lung cancer (Phase 2), and a multimodality imaging dataset obtained from multiple patients (Phase 3). Published in March 2020, IBSI-1 established consensus reference values for 169 commonly used radiomic features, providing an essential benchmark for quality assurance, methodological consistency, and reproducibility in radiomics research. Collectively, these standardization efforts represent a crucial step toward the reliable integration of radiomics and AI into routine clinical practice, where reproducibility and external validation are prerequisites for widespread adoption [164].

Consequently, artificial intelligence is expected to play a central role in the future of coronary imaging. By enabling automated plaque characterization, quantitative risk assessment, and integration with advanced CT technologies, AI has the potential to significantly improve the non-invasive identification of vulnerable plaques and the personalized management of patients with coronary artery disease.

## 8. Limitations of CCTA in Vulnerable Plaque Detection

Although coronary computed tomography angiography (CCTA) has emerged as a valuable non-invasive modality for the evaluation of coronary artery disease and plaque morphology, several limitations remain in the detection and characterization of vulnerable plaques. These limitations are primarily related to spatial resolution, imaging artifacts, and the inherent complexity of plaque biology. Understanding these constraints is essential for the accurate interpretation of CCTA findings and for integrating imaging results into clinical decision-making.

### 8.1. Limited Spatial Resolution

One of the principal limitations of CCTA is its limited spatial resolution compared with invasive imaging techniques, such as optical coherence tomography. While modern CT scanners provide spatial resolutions of approximately 0.4–0.6 mm, this remains insufficient to directly visualize microscopic plaque structures such as the fibrous cap thickness, which is a key determinant of plaque vulnerability. Thin-cap fibroatheromas, defined by fibrous caps thinner than approximately 65 μm, therefore cannot be directly identified by conventional CCTA. Instead, CT imaging relies on indirect imaging markers, such as low-attenuation plaque and the napkin-ring sign, which may serve as surrogate indicators of vulnerable plaques [165,166,167,168,169].

### 8.2. Blooming Artifacts from Calcifications

Heavily calcified plaques represent another important limitation in CCTA interpretation. Dense calcifications may produce blooming artifacts, which cause an apparent enlargement of calcified regions and obscure adjacent plaque components. This phenomenon can make it difficult to accurately evaluate the underlying plaque composition and may lead to overestimation of luminal stenosis or misclassification of plaque type. As a result, the detection of non-calcified or lipid-rich components within heavily calcified segments remains challenging [170].

New technologies such as dual energy CT, spectral CT or photon-counting detector computed tomography, associated with specific software solutions, manage to remove most of these artifacts and provide adequate image quality [72,74,171].

### 8.3. Motion Artifacts and Image Quality

Image quality in CCTA is highly dependent on cardiac motion and heart rate. Motion artifacts, particularly in patients with elevated or irregular heart rates, may reduce image quality and limit accurate plaque assessment. Even within this evolving technological landscape, recent innovations in CT imaging have substantially improved acquisition speed and image quality. Modern scanners now feature gantry rotation times ranging from 0.23 to 0.35 s and support wide detector coverage with a pitch of up to 16 cm, enabling imaging of the entire heart within a single rotation. These advancements are further complemented by improved reconstruction algorithms and post-processing software designed to minimize motion-related degradation and reduce blooming artifacts [144,172,173,174].

The study conducted by Mergen et al. [175], utilizing a dual-source photon-counting detector CT system, provides further evidence supporting these technological improvements. Their results demonstrated a significant reduction in both motion and blooming artifacts, particularly in coronary segments containing previously implanted stents. Additionally, the use of dual-energy CT enabled image reconstructions with an ultrathin slice thickness of approximately 0.2 mm, thereby enhancing vessel edge definition and improving visualization of the in-stent lumen.

### 8.4. Variability in Plaque Attenuation Measurements

Quantitative evaluation of plaque composition using CT attenuation values may also be influenced by several technical factors. Variations in contrast enhancement, reconstruction algorithms, tube voltage, and scanner type can affect attenuation measurements within coronary plaques. These factors may introduce variability in the assessment of low-attenuation plaques and may limit the reproducibility of plaque characterization across different imaging platforms [40,176,177,178].

Although they cannot be completely avoided, these limitations can be reduced by standardizing the image acquisition protocol, by using smart injectors that calculate both the volume of contrast material based on body weight and the injection flow, and by using new generation software solutions. A step forward is represented by integrated artificial intelligence solutions. These, by performing segmentations and choosing significant regions of interest (ROI), can improve the quality of the radiological report.

### 8.5. Radiation Exposure

Despite significant reductions in radiation dose with modern CT technologies, exposure to ionizing radiation remains an important consideration in CCTA. Although contemporary scanning protocols have substantially lowered radiation doses, cumulative exposure may still be relevant in patients requiring repeated imaging for disease monitoring [179,180,181].

As with the previous limitations, radiation exposure in CCTA may benefit from the introduction of new technologies. Benz et al. [182] obtained diagnostic-quality images in the majority of coronary segments (98.4%) using a 256-slice scanner. The amount of contrast medium administered was in the range of 25–40 mL adjusted for body weight at a rate of the injection speed of 3.5–5 mL/s, while the radiation dose was 0.4–0.6 mSv.

## 9. Future Perspectives

Ongoing technological developments are expected to address some of these limitations. Advances such as spectral CT, photon-counting detector CT, and artificial intelligence-based image analysis may improve plaque characterization, reduce imaging artifacts, and enhance the detection of vulnerable plaque features.

One of the most promising technological developments in cardiac CT is photon-counting detector CT (PCCT). Unlike conventional energy-integrating detectors, photon-counting detectors measure the energy of individual X-ray photons and categorize them into different energy bins. This technology offers several advantages, including improved spatial resolution, reduced image noise, and enhanced spectral separation. In the context of coronary plaque imaging, PCCT may allow improved visualization of small coronary structures, microcalcifications, and subtle differences in plaque composition. The higher spatial resolution of photon-counting CT may also improve the assessment of non-calcified plaques and potentially enable more precise identification of lipid-rich components associated with plaque vulnerability.

The continued development of spectral CT techniques is expected to further improve tissue characterization in coronary imaging. Spectral imaging enables the differentiation of materials based on their energy-dependent attenuation profiles, allowing improved discrimination between plaque components such as lipid, fibrous tissue, and calcium. Future spectral CT applications may include quantitative material decomposition, improved detection of lipid-rich plaques, and enhanced evaluation of intraplaque contrast enhancement related to neovascularization and inflammation. These capabilities could significantly improve the identification of plaques at high risk of rupture.

Beyond plaque morphology, future CCTA applications will increasingly incorporate functional and hemodynamic evaluation of coronary lesions. Techniques such as CT-derived fractional flow reserve (FFR-CT) allow non-invasive estimation of the physiological significance of coronary stenoses.

In addition, emerging computational approaches may enable the assessment of wall shear stress and biomechanical forces acting on coronary plaques. These parameters are believed to play a key role in plaque progression and rupture and may therefore contribute to more accurate prediction of plaque instability.

Ultimately, the integration of advanced CT technologies, artificial intelligence, and multimodality imaging is expected to support a shift toward precision cardiovascular medicine. By combining detailed anatomical information with molecular, functional, and computational insights, future imaging strategies may enable earlier detection of vulnerable plaques and more personalized management of patients with coronary artery disease.

As these technologies continue to evolve and undergo clinical validation, CCTA is likely to play an increasingly central role in the non-invasive assessment of coronary atherosclerosis and the prevention of acute coronary events.

Nevertheless, CCTA findings should always be interpreted within the broader clinical context and, when necessary, complemented by other imaging modalities for comprehensive plaque assessment.

## 10. Conclusions

Coronary computed tomography angiography has evolved from a technique primarily used to assess luminal stenosis into a powerful non-invasive modality capable of providing comprehensive information about coronary atherosclerosis. In recent years, significant progress has been made in the identification of imaging biomarkers associated with plaque vulnerability, allowing CCTA to contribute not only to the diagnosis of coronary artery disease but also to the evaluation of plaque composition and risk stratification.

Several CT-derived features, including low-attenuation plaque, positive remodeling, spotty calcifications, and the napkin-ring sign, have been consistently associated with vulnerable plaques and an increased risk of adverse cardiovascular events. The identification of these high-risk plaque characteristics enables earlier recognition of patients who may benefit from intensified preventive strategies, including aggressive lipid-lowering therapy and closer clinical monitoring.

Advances in quantitative plaque analysis, spectral CT imaging, and artificial intelligence-based image processing are further expanding the capabilities of CCTA in plaque characterization. These technologies allow more precise assessment of plaque burden and composition, facilitate automated analysis of large imaging datasets, and may improve the prediction of future cardiovascular events.

Despite these advances, several limitations remain, including restricted spatial resolution for the evaluation of microscopic plaque features, imaging artifacts related to calcifications, and the limited ability of conventional CT to assess biological processes such as inflammation. For this reason, multimodality imaging approaches combining CCTA with functional or molecular techniques may further enhance the evaluation of plaque instability.

Overall, CCTA represents a key component of modern cardiovascular imaging and plays an increasingly important role in the non-invasive detection of vulnerable plaques and cardiovascular risk stratification. Continued technological developments and integration with advanced computational methods are expected to further improve the diagnostic and prognostic value of CCTA, ultimately contributing to more personalized management and improved outcomes for patients with coronary artery disease.

## Figures and Tables

**Table 1:** Hemodynamic Factors Influencing Coronary Plaque Formation and Vulnerability.

Hemodynamic Factor	Mechanism	Effect on Vessel Wall	Clinical/Imaging Implications
Low wall shear stress (WSS) [25,31,32,33]	Reduced tangential force of blood flow on the endothelial surface	Promotes endothelial dysfunction, lipid accumulation, and inflammatory cell infiltration	Associated with plaque initiation and progression
Oscillatory shear stress [25,32,33,34]	Reversal or fluctuation of blood flow direction during the cardiac cycle	Stimulates pro-inflammatory endothelial phenotype and oxidative stress	Frequently observed in arterial bifurcations and curved segments
Turbulent blood flow [31,35,36]	Irregular flow patterns caused by abrupt changes in vessel geometry	Increases endothelial injury and promotes atherogenesis	Occurs near stenoses, bifurcations, and abnormal vessel origins
Abnormal vessel angulation [26,35,36,37]	Acute take-off angles or sharp vessel curvature	Alters local shear stress distribution and creates zones of low flow	Associated with localized plaque formation
Bifurcation geometry [26,35,38]	Complex flow patterns at branching points	Produces heterogeneous shear stress distribution on vessel walls	Plaques frequently develop along lateral walls of bifurcations
Positive vascular remodeling [24,25,26,31,39,40]	Compensatory enlargement of the vessel wall in response to plaque growth	Maintains lumen diameter despite increasing plaque burden	Often associated with high-risk plaques on CCTA
Extrinsic vessel compression [24,25,41,42]	Mechanical compression from surrounding structures (e.g., myocardial bridging)	Alters coronary flow dynamics and increases proximal wall stress	May contribute to proximal plaque formation

**Table 2:** Comparison between IVUS, OCT and NIRS in coronary plaque characterization.

Imaging Modality	Type of Information	Main Plaque Features Detected	Spatial Resolution	Main Advantages	Main Limitations
Intravascular ultrasound (IVUS) [56,57,58]	Cross-sectional structural and morphological imaging of the coronary artery and vessel wall	Plaque burden and volume; positive remodeling; plaque morphology; vessel wall architecture; overall extent of atherosclerotic disease	Moderate; lower than OCT	Enables visualization of the entire vessel wall; provides relatively deep tissue penetration; allows quantitative assessment of plaque volume, vessel dimensions, and remodeling; useful for evaluating the overall extent of coronary atherosclerosis	Limited ability to identify fine plaque microstructures; lower resolution than OCT; less accurate for assessing fibrous-cap thickness and other microscopic features of vulnerable plaques
Optical coherence tomography (OCT) [57,58,59]	High-resolution morphological and microstructural imaging of coronary plaques	Fibrous-cap thickness; thin-cap fibroatheroma; macrophage infiltration; microchannels; plaque rupture; coronary calcifications	Very high; up to ten times higher than IVUS	Provides detailed visualization of plaque microstructure; accurately measures fibrous-cap thickness; highly effective for identifying thin-cap fibroatheromas and plaque rupture; may be combined with angiographic coregistration and artificial intelligence–based analysis	Limited tissue penetration compared with IVUS; assessment of the entire plaque burden and deeper vessel-wall structures may be incomplete
Near-infrared spectroscopy (NIRS) [60,61]	Spectroscopic and compositional assessment of coronary plaques	Lipid-rich plaques; lipid-core burden; plaques with large lipid cores associated with an increased risk of future vascular events	Limited anatomical spatial resolution; primarily provides compositional rather than detailed structural information	Specifically detects lipid-rich plaques; provides information about plaque composition; can be combined with IVUS to integrate lipid-core assessment with structural and morphological imaging	Provides limited information regarding plaque morphology and vessel-wall architecture when used alone; commonly requires combination with IVUS for comprehensive plaque assessment

**Table 3:** Comparison between CCTA, MRI and PET in coronary plaque characterization.

Imaging Modality	Type of Information	Main Plaque Features Detected	Spatial Resolution	Main Advantages	Main Limitations
Coronary CT Angiography (CCTA) [62,63,68,69,70]	Structural/anatomical	Plaque morphology, plaque burden, calcified and non-calcified plaque, low-attenuation plaque, positive remodeling, napkin-ring sign, spotty calcifications	High (≈0.4–0.6 mm)	Excellent visualization of coronary lumen and vessel wall; detection of high-risk plaque features; rapid acquisition; widely available	Limited ability to assess plaque microstructure (e.g., fibrous cap thickness); blooming artifacts from calcifications; ionizing radiation
Cardiac Magnetic Resonance Imaging (CMR/MRI) [91,92,93]	Structural and tissue characterization	Vessel wall structure, lipid-rich necrotic core, intraplaque hemorrhage, fibrous tissue composition	Moderate (≈1–2 mm)	Superior soft-tissue contrast; no ionizing radiation; allows simultaneous evaluation of myocardial perfusion, viability, and function	Limited spatial resolution for coronary arteries; long acquisition time; motion artifacts; limited availability for coronary plaque imaging
Positron Emission Tomography (PET) [66,94,95]	Functional/molecular	Plaque inflammation (^18F-FDG uptake), active microcalcification (^18F-NaF uptake), metabolic activity	Low (≈4–5 mm)	Provides metabolic and inflammatory information; detects biological processes underlying plaque instability	Limited anatomical detail; relatively low spatial resolution; myocardial tracer uptake may interfere with coronary imaging; high cost
Hybrid Imaging (PET/CT, PET/MRI) [96]	Combined anatomical and molecular imaging	Plaque morphology + inflammation + microcalcification	Variable	Integrates structural and biological information; improved risk stratification	Limited availability; higher cost; complex acquisition protocols

**Table 4:** High-risk plaque features identified on Coronary CT Angiography.

Imaging Feature	CT Definition	Pathophysiological Correlate	Clinical Significance
Low-attenuation plaque [66,100,101,102]	Plaque attenuation typically <30 HU within non-calcified plaque	Lipid-rich necrotic core	Strong predictor of plaque vulnerability and future acute coronary syndromes
Positive remodeling [103,104,105,106]	Remodeling index (RI) > 1.1 (lesion vessel diameter/reference diameter)	Inflammatory-mediated vessel wall expansion	Associated with unstable plaques and increased risk of major adverse cardiac events
Napkin-ring sign [62,108,109,110,111]	Central low-attenuation core surrounded by a higher-attenuation rim	Thin-cap fibroatheroma with large lipid core	Highly specific marker of vulnerable plaque and future coronary events
Spotty calcifications [66,68,103,104,120]	Small focal calcifications < 3 mm within non-calcified plaque	Early microcalcification within inflamed plaques	Marker of plaque instability and progression
Large plaque burden [113,126]	Plaque occupying > 70% of vessel cross-sectional area	Advanced atherosclerotic disease	Associated with increased risk of plaque rupture
Low-attenuation plaque volume [9,14,122,128]	Increased volume of plaque with attenuation < 30–60 HU	Lipid accumulation and necrotic core expansion	Predictor of adverse cardiovascular events
High-risk plaque phenotype [62,103,104,121,122]	Presence of ≥2 high-risk features	Combination of structural instability markers	Significantly higher risk of acute coronary syndrome
Perivascular fat attenuation index (FAI) [97,98,112,113]	Increased CT attenuation of perivascular adipose tissue	Vascular inflammation	Emerging biomarker of coronary inflammation and risk prediction

**Table 5:** Major clinical trials linking CCTA high-risk plaque features with cardiovascular events.

Study	Year	Study Population	Key Imaging Findings	Main Conclusions
SCOT-HEART [124]	2015/2018	4146 patients with suspected CAD	CCTA identified both obstructive CAD and high-risk plaque features	CCTA improved diagnostic certainty and was associated with a reduction in myocardial infarction during follow-up
PROMISE [126]	2015	10,003 patients with stable chest pain	CCTA allowed detection of non-obstructive CAD and plaque burden	Plaque burden identified by CCTA predicted major adverse cardiovascular events better than stenosis alone
ICONIC [128]	2018	234 patients with serial CCTA examinations	Low-attenuation plaque and positive remodeling strongly associated with future ACS	High-risk plaque features on CCTA predict culprit lesions responsible for acute coronary syndromes
PARADIGM [129]	2017	2252 patients undergoing serial CCTA	Quantitative plaque analysis showed progression of non-calcified plaque over time	Statin therapy associated with reduced plaque progression and increased plaque calcification (stabilization)
ROMICAT II [127]	2013	1000 patients presenting with acute chest pain	CCTA enabled early detection of coronary plaque and luminal stenosis	CCTA improved triage and reduced hospital stay in emergency settings
NAPKIN-RING SIGN studies [99,103,105]	2014–2019	Various prospective cohorts	Presence of napkin-ring sign strongly associated with future ACS	Napkin-ring sign considered one of the most specific CT markers of vulnerable plaque
CRISP-CT Study [97]	2018	3912 patients undergoing CCTA	Perivascular Fat Attenuation Index (FAI) used as marker of coronary inflammation	Increased FAI associated with higher risk of all-cause and cardiac mortality

## Data Availability

The original contributions presented in this study are included in the article. Further inquiries can be directed to the corresponding author.
